# Lost in transduction: Critical considerations when using viral vectors

**DOI:** 10.3389/fcell.2022.1080265

**Published:** 2023-01-05

**Authors:** William C. Hines, William C. Hines

**Affiliations:** ^1^ Sandia National Laboratory, Albuquerque, NM, United States; ^2^ Department of Biochemistry and Molecular Biology, University of New Mexico School of Medicine, University of NM, Albuquerque, NM, United States

**Keywords:** lentiviral vectors, viral transduction, multiplicity of infection (MOI), scientific reproducibility, cell barcoding

## Abstract

The application of retroviral vectors in the laboratory requires considerations that often go overlooked but are often easy to circumvent. Here, we discuss the relationship between the observed transduction efficiency of a cell population and per-cell viral insertions—and describe how differential cell-type susceptibilities can confound results. We consider the math underlying this problem and review an alternative approach to the commonly used “multiplicity of infection” (MOI) method of titering and using viral vectors in the biomedical research laboratory.

## Introduction

In laboratory science, problems often arise in seemingly mundane experimental details (Hines et al., 2014; Bradbury and Pluckthun, 2015; Freedman, 2015; Madsen and Bugge, 2015; Schonbrunn, 2015; Lithgow et al., 2017). Given the increasingly more complex tools used in biomedical research, accurately performing and interpreting sophisticated experiments requires more specialized expertise and even more attention to every small detail. Yet, the enormous competition of academic scientists to publish before others, and the need for more papers to obtain grants and positions, appears to present investigators with less and less time to absorb and analyze each step they take in performing published—or even seemingly routine—procedures. The result is confusion, problems with reproducibility, or worse. Moreover, even widely used tools and techniques frequently contain subtle implicit details that can profoundly affect results, not all of which are obvious, generally understood, or appreciated—as we all have likely and frustratingly encountered.

Here, we consider viral transduction–a seemingly simple technique used widely in many biomedical laboratories worldwide that supports countless publications. On the surface, the task of using viruses to deliver foreign genetic material to cells in a laboratory contains only a few simple steps: Virus is bought or created, viral particles are applied to cultured cells, and transduced cells are selected for further analysis.

## Complexities of viral transductions interfere with quantitative assessments

**FIGURE 1:**
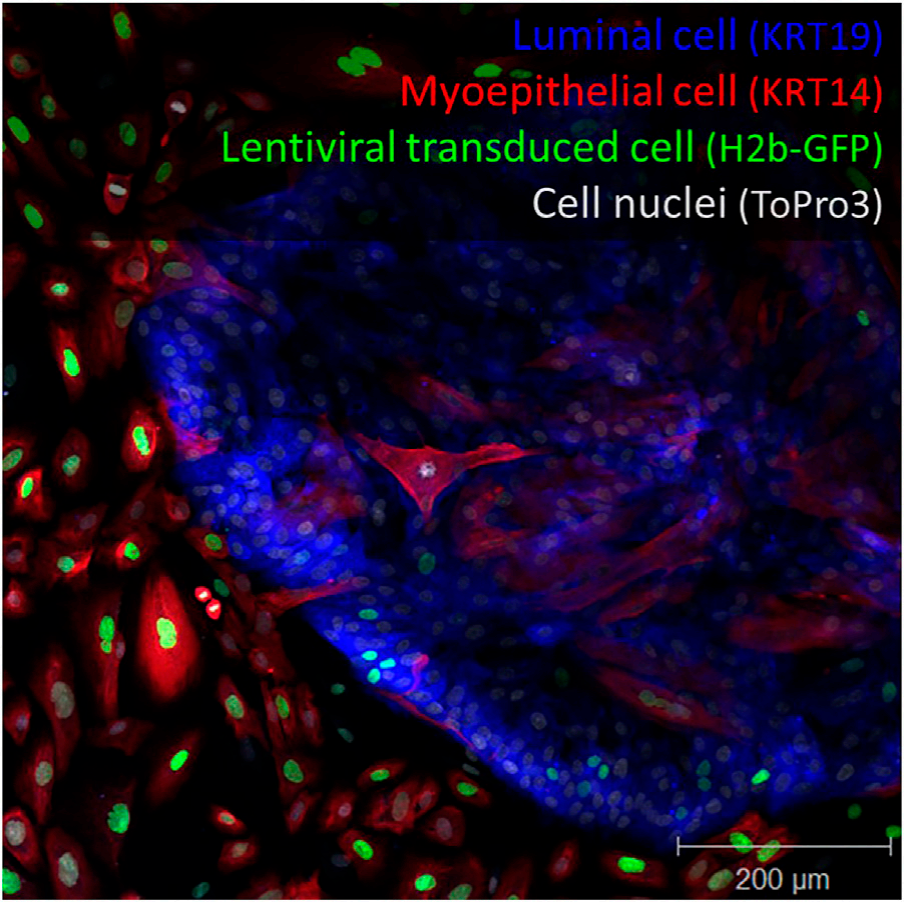
Lentiviral transduction bias. Primary breast culture containing luminal cells (keratin 19^+^, blue), myoepithelial cells (keratin 14^+^, red) transduced with a histone 2-beta GFP encoded lentivirus (H2b-GFP, green). Staining reveals imbalanced cell transduction with respect to cell type. Nuclei are counterstained with ToPro3 dye. Scale = 200 μm.

Using a similar lentiviral strategy several years ago, we were surprised to discover that cells in our primary breast cultures (derived from reduction mammoplasty) were not being evenly transduced when treated with lentiviruses (Hines et al., 2015). We found luminal epithelial cells (LEPs) in these cultures to be significantly more resistant to viral transduction than their myoepithelial counterparts (MEPs). This bias could be easily observed using fluorescently tagged virus. Figure 1 shows the clear demarcation zone in these primary tissue-derived cultures. We searched the literature to see if others had reported data related to this dramatic observation. Relevant data were scant. However, the potential ramifications were instantly clear: The selective transduction we observed would distort conclusions if the bias went unnoticed, as we suspected it had. We wondered whether this bias could explain why transductions—and current breast tumor models that have depended on oncogenic transformation—produce cell lines with predominantly basal/myoepithelial-like phenotype.

The importance of answering this question and the need to balance transductions between cell types set off a multi-year endeavor to characterize this finding, uncover a mechanism, and develop a solution. Importantly, we could show that the bias extended beyond primary normal cells and carried over to the existing and commonly used breast cancer cell lines. After trying many different methods and procedures to balance transduction between the two epithelial cell types, we found the answer lay in differential viral binding due to glycosaminoglycans (GAGs) on the cell’s surface. Our solution was to treat the cells (or virus) liberally with neuraminidase. This evened the odds, and the myoepithelial and luminal cell lineages in these primary cultures were evenly transduced—and this ultimately dictated the types and properties of the cell lines that emerged (Hines et al., 2015).

This exercise, and the many experiments required to solve the problem, persuaded us to question whether we were considering viral titer and measuring the multiplicity of infection (MOI) correctly*.* In many product manuals and online resources, MOI is often defined as the number of virus particles per cell transduced. However, when transducing heterogeneous cell populations, as we were trying to do, MOI strictly defined this way made little sense and is, quite frankly, misleading (which will not surprise virologists—which we are not). The different cell types present in these mixed primary cultures were exposed to identical virus preparations and doses by their very nature of coexisting in a single dish. Nevertheless, we found the transductional efficiencies between cell lineages to be dramatically different. Others have also cautioned that the complexities of viral transductions are not accurately captured by the term “MOI,” and have suggested that this term is used as a quantitative measure and should be discontinued (Shabram and Aguilar-Cordova, 2000). Given the current climate and renewed emphasis on scientific reproducibility, perhaps we should revisit this proposal. The conditions of the experimental systems can (and do) have profound effects on infectivity. Dulbecco and Vogt, who used the Poisson distribution to explain mechanisms of poliovirus transduction in 1954, modified the equation by inserting a constant that they defined as the “efficiency of the system.” They used this constant to account for variability and unexplained differences in transduction -much like that we observed between luminal and myoepithelial cells (Dulbecco and Vogt, 1954). Unfortunately, this ‘constant’ and the emphasis on experimental conditions dropped from textbook definitions and product manuals. As a result, much confusion surrounding the use of MOI has ensued (Shabram and Aguilar-Cordova, 2000), especially for those outside virology circles.

Early in our study, our results made us question whether luminal cells even contained the proper cell “machinery” that makes them permissive to lentivirus transduction. We used flow cytometry to precisely count the number and types of different cell types transduced by serial dilutions of the virus, and we fit these results to the Poisson distribution. We discovered that despite the significant quantitative differences observed between cell types, both types of cells were in fact being transduced in a similar qualitative fashion. That is, both cell types fit the Poisson probability mass function when we correctly accounted for the “efficiency” of the system. Luminal cells could be transduced; it just took much more virus. When we accounted for these differences, we found that their transduction patterns were identical to myoepithelial cells!

The above analysis led us to reason that when cells are exposed to virus, two probabilities at play dictate whether the cells become transduced. There is: 1) the chance that a cell will encounter a viral particle (which is explained by the Poisson equation) and 2) the binomial probability that, if a cell encounters a viral particle, it will be transduced. By combining these two probabilities, we justified using a new term, the “effective cell transducing volume” (ECTV) (Hines et al., 2015). It is calculated by: $={Vol}_{50}/\left(N\;\right)*1/0.693$ , where Vol_50_ is the volume of viral stock needed to achieve 50% cell transduction, *n* = number of cells transduced, and 0.693 is the negative natural log of cells transduced, i.e., 50%, calculated as -ln (0.5).

ECTV represents the small volume of virus that, when applied to a cell, transduces it with 100% efficiency. ECTV is not difficult to calculate and is helpful in practice because it is defined in terms of virus stock volume—rather than some measure of particle concentration. Because ECTVs are functionally defined, they are not solely a characteristic of the viral preparation but reflect the entire system. Transductions, and thus ECTVs, are influenced by cell type and experimental conditions, including composition and volumes of the medium used, time, temperature, presence of serum, etc. As such, ECTV ratios calculated for the same cells- but under different conditions-reflect the effects the conditions have on that particular cell type under the given experimental context. For example, when we tested the effects of polybrene on three cell lines (polybrene is a polymer used to enhance viral transductions), we found it indeed improved transductions, but the effects varied widely. At equivalent doses, one cell line was improved by as much as 26-fold (MDA-MB-468), another 11-fold (MCF-7), and yet another by only 3-fold (MDA-MB-231). If we had not used ECTV values for this analysis, the interpretation would have been much more complicated due to the vastly different transduction efficiencies exhibited by these cells and conditions. Using ECTVs reduced the analysis to a single value and clarified results.

## Another complicating factor: Viral integrations

**FIGURE 2:**
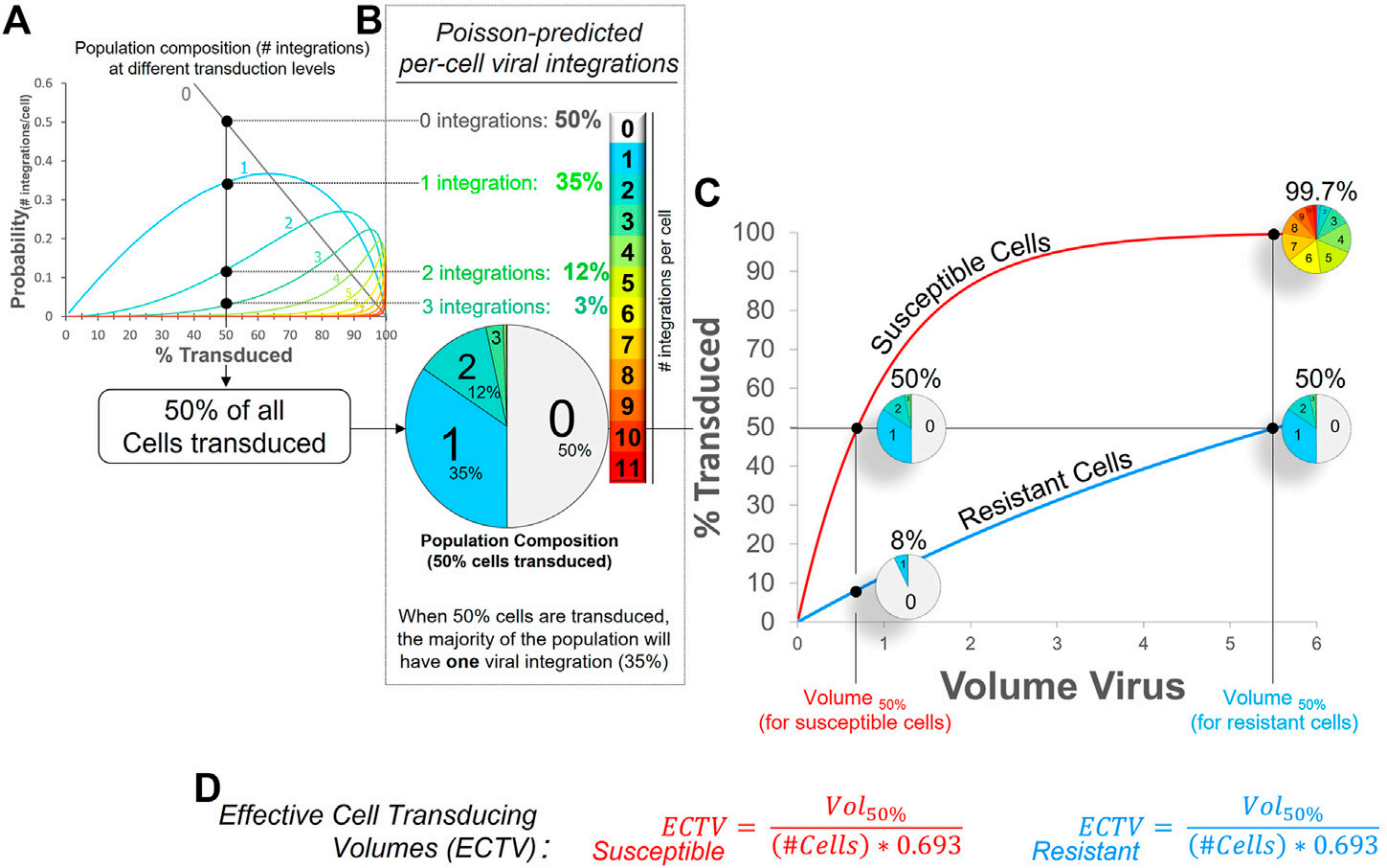
Essence of lentiviral transductions and ECTV calculation. **(A)** Poisson mass distribution function: Shown is the predicted relationship between overall transduction efficiency (% cells transduced) and the expected cell fractions containing between one and eleven viral integrations (light blue to red traces). **(B)** Pie chart illustrating, when 50% of all cells in a population are transduced, the predicted proportion of cells in that population that have zero, one, two, or three viral integrations (per cell). **(C)** Poisson-predicted viral transduction of two cell types displaying an 8-fold difference in viral susceptibility. Without a method to balance transductions between coexisting cell types, susceptible cells will always, at all viral doses, have 8× more viral integrations than the resistant cells, as illustrated by the small pie charts. **(D)** The volume of virus stock required to achieve a defined transduction level can be predicted by determining the respective ECTV (Effective Cell Transducing Volume) for each cell type. At 50% transduction efficiency, the ECTV is defined by the volume of viral stock (that produced 50% cell transduction, Vol_50%_), divided by the product of the number of cells and the natural log of 0.5, i.e., (# cells × 0.693). For example, if 0.8 ul of virus was required to transduce 50% of 100,000 cells, the ECTV for this cell type (under these conditions) calculates to 0.8 ul/-(100,000*ln 0.5); which is 11.54 picoliters (pL). Once this ECTV is known, one can more reliably predict the amount of virus required to transduce a different fraction of cells; e.g., transducing 85% of cells under the same conditions will require 2.19 ul of virus stock (11.54 E^−6^ ul*100,000*-ln0.15).

Multiple integrations are yet another complicating factor important to consider, especially when modeling cancer—and also when using barcoding libraries (Krutzik and Nolan, 2006; Bhang et al., 2015). When treated with common laboratory lentiviruses, cells are permissive to multiple viral particles and will be transduced more than once. This, in turn, leads to the accumulation of viral integrations in the cell’s genome. The relationship between lentiviral dose, transduction efficiencies, and multiple viral integrations is explained by the Poisson distribution and is illustrated in Figure 2A. For example, when half of all cells are transduced, the fractions of cells that have 0, 1, 2 or 3 integration constitute 50%, 35%, 12%, and 3% of the population, respectively (Figure 2B). The caveat is that these differences are difficult to discern in live cells at present, but they can be evaluated in fixed cells by measuring DNA copy numbers—or barcodes. Not surprisingly, we showed that cells with more integrations indeed have higher levels of transgene expression (Hines et al., 2015), and with each genomic insertion, there is an increasing risk that essential endogenous genes can be disrupted. The very real risk is that this may lead to clonal expansion or other artifacts. Furthermore, these differences are hidden in typical graphs of transduction efficiencies (Figure 2C). What appears at first glance as a two-fold difference between cell types (99.7% vs. 50% at the highest viral dose), is in fact a much bigger difference when one considers all the viral integrations these cells acquire (small pie charts in Figure 2C). If one could sum up all integrations within these cells, the fold difference between cell types is simply the difference in their ECTVs. In this case, 8-fold–a difference that exists at all viral doses. A graphical depiction of how these ECTVs are calculated is shown in Figure 2D.

For the sake of scientific reproducibility, perhaps less attention should be placed on viral dose (MOI) (Shabram and Aguilar-Cordova, 2000), and more on the transduction efficiencies obtained in a given experiment. We submit that the ECTV method may provide a useful alternative.

## Materials and methods

### Primary cell staining

Immunofluorescence was performed on primary cultures of human mammary epithelial cells (HMEC) transduced with H2b-EGFP lentivirus as previously described (Hines et al., 2015). Briefly, cultures were treated with 4% paraformaldehyde for 5 min at 23°C, followed by 4% formaldehyde/0.1% saponin for 5 min at 23°C. Samples were subsequently incubated for 20 min in wash buffer (0.1% saponin/10% goat serum in PBS), and incubated with primary antibodies (keratin 19 and keratin 14) diluted in wash buffer at 1:400 dilution ratios. Samples were incubated overnight at 4°C. Following the primary antibody incubations, samples were washed and incubated with anti-mouse and anti-rabbit secondary antibodies, respectively conjugated with Alexafluor 405 and 594 (Thermo), diluted 1:400 in wash buffer. After 1 h incubation at 23°C, samples were rinsed in PBS and their nuclei counterstained with ToPro3 (Thermo). Coverslips were mounted with Fluoromount G (Southern Biotech).

### ECTV and poisson modeling

Effective Cell Transducing Volume (ECTV) is calculated by:

$$ECTV=\frac{{Vol}_{50}}{\left(N\;\right)}*\frac{1}{0.693}$$

Where Vol_50_ is the volume of viral stock needed to achieve 50% cell transduction, N is the number of cells transduced, and 0.693 is the negative natural log of cells transduced, i.e., 50%, calculated as -ln (0.5). Figure 2 is a graphical representation of the Poisson Probability mass function, that describes the probability, P(x), of any given cell receiving “x” virus particles, where lambda (λ) is the average number of virus particles per cell.

$$P_{\left(x\right)}=\frac{\lambda^{x}\cdot e^{-\lambda }}{x!}$$

## Data Availability

The original contributions presented in the study are included in the article/supplementary files, further inquiries can be directed to the corresponding author.
